# Joint Profiles and Dimension-Level Networks of Parental Psychological Control and Problematic Short-Video Use Among Chinese Adolescents

**DOI:** 10.3390/bs16101893

**Published:** 2026-10-13

**Authors:** Yan Yang, Yuchi Zhang

**Affiliations:** 1 School of Education Science, Xinjiang Normal College (Xinjiang Education Institute), Urumqi 830000, China; 2 Department of Educational Technology, Faculty of Education, Jiangsu Normal University, Xuzhou 221116, China

**Keywords:** problematic short-video use, parent psychological control, latent profile analysis, network analysis

## Abstract

Problematic short-video use (PSVU) is a pervasive public health concern among adolescents, yet its relationship with parental psychological control has typically been examined through variable-centered approaches that treat this relationship as uniform and combine paternal and maternal control. The present study therefore examined joint configurations of paternal and maternal psychological control and PSVU, along with their dimension-level interrelations. Using a two-stage design, latent profile and psychometric network analyses were applied to 2634 Chinese adolescents. Subgroups were first identified based on paternal psychological control, maternal psychological control, and PSVU; dimension-level network structures were then compared across profiles to examine differences in the associations between parental control and PSVU dimensions. The results revealed four distinct profiles: a High-Control/High-Dependence profile, a High-Control/Low-Dependence profile, a Low-Control/Low-Dependence profile, and an Average-Control/Moderate-Dependence profile. The high-control, low-dependence profile reported higher control but significantly lower PSVU. Network comparisons indicated that the high-control, high-dependence profile exhibited significantly stronger global network connectivity than the high-control, low-dependence profile, whereas direct bridge pathways linking parental control to PSVU dimensions did not differ significantly between the two profiles. These findings suggest that the parental control–PSVU relationship is neither uniform nor direct, underscoring the value of integrating person-centered and network approaches.

## 1. Introduction

With the widespread use of smartphones among adolescents, problematic short-video use (PSVU) has become a global public health concern (Z. H. Jiang et al., 2026). TikTok has proliferated in recent years as a dominant force in the short-video app landscape. By 2021, the expansion rate of its user base in the United States more than doubled the combined growth of traditional social media platforms, including Twitter, Instagram, and Facebook (Schellewald, 2021). Notably, this short-video application holds immense appeal for younger demographics; in 2020, adolescents aged 10–19 years accounted for 32.5% of all active users (Clement, 2026; McCashin & Murphy, 2022). As a form of problem behavior, PSVU is closely associated with severe depression, anxiety, and related difficulties among adolescents (Qu et al., 2024; Zhu et al., 2024). Family factors, such as parental psychological control, are closely linked to adolescent problem behaviors (Romm & Metzger, 2018).

However, several gaps exist in the literature. First, although PSVU is increasingly recognized as a distinct form of problematic digital behavior with mechanisms that differ from generalized Internet or smartphone use, it is often subsumed under broader problematic technology use, leaving its specific correlates insufficiently examined (Liao, 2024; Zhang et al., 2026). Second, research on parental psychological control and adolescents’ problematic digital media use has predominantly relied on variable-centered approaches that estimate average associations across the population, treating this relationship as uniform (Q. Q. Li & Liu, 2025). Such approaches obscure potential heterogeneity in how this relationship is structured across adolescents, and they have generally combined paternal psychological control (PPC) and maternal psychological control (MPC) rather than distinguishing them. Third, insufficient attention has been paid to population heterogeneity within Chinese adolescent samples, limiting the ability to account for culturally embedded family processes.

Accordingly, the present exploratory study addresses three research questions. First, do PPC, MPC, and PSVU form heterogeneous joint profiles among Chinese adolescents? Second, among profiles that differ in PSVU outcomes, do the dimension-level networks linking parental control to PSVU dimensions differ in structure? Third, if such structural differences exist, where do they lie? To address these questions, the present study adopted a two-stage design, first employing latent profile analysis (LPA) to identify heterogeneous subgroups and then applying psychometric network analysis to compare the dimension-level relational structures across these subgroups.

## 2. Literature Review

### 2.1. Problematic Short-Video Use and Parental Psychological Control

Unlike generalized Internet or smartphone use, Internet addiction, or Internet gaming disorder, which typically involve more goal-directed activities and clearer session boundaries, PSVU is characterized by frictionless, algorithm-driven consumption through immersive vertical scrolling and blind-box instant gratification, accompanied by pronounced withdrawal reactions upon cessation (Liao, 2024; Liu et al., 2026; Zhang et al., 2026). Short, highly stimulating content, infinite scrolling, and personalized recommendations provide continuous variable-ratio reinforcement, which may foster mindless absorption, temporal distortion, rapid tolerance, and involuntary viewing patterns (Galanis et al., 2024; A. Jiang et al., 2025; Yang et al., 2024). These behavioral features and distinct factor structures suggest that PSVU is a distinct form of problematic digital behavior. This hyperstimulating format is especially hazardous for adolescents, as adolescence is a developmental period marked by immature self-regulatory capacities and heightened, hormonally driven sensitivity to immediate rewards; adolescence constitutes a window of particular vulnerability to this novel behavioral addiction (Liu et al., 2026). Excessive engagement with short videos has been robustly associated with cognitive, behavioral, and emotional impairments, including diminished sustained attention, academic procrastination, and increased depressive symptoms (Chen et al., 2023; S. Li et al., 2026; Qu et al., 2024; Xie et al., 2023; Zhu et al., 2024), highlighting PSVU as an important developmental concern.

Parental psychological control, defined as intrusive and manipulative practices directed at adolescents’ internal psychological world through guilt induction, love withdrawal, and authority assertion, represents a theoretically central family predictor of problematic adolescent behaviors. Grounded in Self-Determination Theory (SDT), such control practices thwart basic psychological needs for autonomy and relatedness, positioning adolescents at a heightened risk of compensatory engagement with instantly gratifying media, such as short-video platforms (Ryan & Deci, 2000). However, whether and how this risk manifests varies substantially across individuals rather than through uniform linear effects across the population. Considerable cross-sectional and longitudinal empirical research has consistently demonstrated that parental psychological control serves not only as a robust predictor of problematic adolescent Internet use but also as a core driver of excessive smartphone use and deviant online behavior (Cetinkaya, 2019; J. Wang & Jia, 2026). Longitudinal evidence indicates that coercive parental psychological control is associated with problematic mobile phone use in adolescents (Q. Q. Li & Liu, 2025). Furthermore, it indirectly catalyzes and solidifies problematic behaviors by provoking psychological reactance or exacerbating emotional distress, such as anxiety and depression (Q. Q. Li & Liu, 2025; Z. Xiong et al., 2026). However, previous studies have rarely focused specifically on PSVU, despite emerging evidence that the psychological patterns associated with short-video consumption may differ from those observed in generalized smartphone or Internet use (Liao, 2024). Furthermore, prior research has largely failed to differentiate between PPC and MPC, thereby limiting our capacity to gain a deeper understanding of these potentially distinct constructs (Q. Q. Li & Liu, 2025).

### 2.2. Person-Centered and Network Approaches

Despite the theoretical importance of differentiating parental psychological control, existing research has almost exclusively relied on variable-centered approaches, including regression, mediation, and moderation models, which assume population homogeneity and yield coefficients representing average effects across the full sample. This assumption is at odds with Developmental Systems Theory (DST), which posits that identical risk exposures can lead to qualitatively different developmental outcomes through complex, bidirectional individual-context interactions, reflecting the principles of equifinality and multifinality (Lerner, 2006). Variable-centered models therefore obscure the subgroup-level heterogeneity that DST predicts. Consistent with this concern, prior work has shown that associations undetectable through traditional regression become apparent once person-centered methods reveal qualitatively distinct subgroups with differentiated adaptation patterns (S. Fang et al., 2023). LPA is well suited to this task, as it identifies subpopulations defined by their joint configurations of parental psychological control and PSVU rather than by average effects estimated across the sample (Spurk et al., 2020). This person-centered stage advances beyond prior variable-centered work by clarifying whether adolescents exposed to comparable family risk diverge into distinct behavioral subgroups.

Identifying such subgroups, however, does not reveal how parental control and PSVU dimensions are interrelated within them. Addressing this requires examining the relational structures operating inside each subgroup (J. Wang & Jia, 2026; Z. Xiong et al., 2026). Traditional latent variable models assume local independence, treating observed indicators as conditionally unrelated once the underlying latent construct is controlled, which precludes examination of how family stressors and dependence dimensions directly interconnect (Guyon et al., 2017). The Network Theory of Psychopathology offers an alternative, conceptualizing constructs as systems of directly interacting components whose patterns of interconnection can be empirically estimated and compared (Borsboom, 2017; Borsboom & Cramer, 2013). Applying this framework within LPA-identified profiles enables examination of whether the dimension-level associative structure differs across subgroups even after accounting for the mean-level differences that define them, information that neither between-profile mean comparisons nor variable-centered correlations can provide. This network stage thus contributes a level of analysis absent from prior research, moving the question from whether parental control predicts PSVU on average toward how the two are structurally associated within distinct adolescent subgroups. Because parental psychological control is theoretically a contextual stressor rather than a behavioral symptom, PPC and MPC were modeled as nodes within a family socialization community and the PSVU dimensions within a behavioral outcome community, following recent network studies that include contextual risk factors as nodes to estimate cross-community associations without equating the theoretical status of the two domains (Contreras et al., 2019; Jones et al., 2021).

### 2.3. Present Study

To comprehensively capture the complex co-occurrence patterns of adolescent PSVU and parental psychological control, it was imperative to enhance the cultural sensitivity of our study by situating these family processes within the specific cultural context of adolescents (Wuyts et al., 2015). In the Chinese society, which is deeply rooted in Confucian filial piety, parental psychological control is exercised through guilt induction and love withdrawal. Parental control is also deeply intertwined with high achievement expectations and child-invested contingent self-esteem (Q. Fang et al., 2022; Wuyts et al., 2015). Under such culturally sanctioned family pressures, adolescents may be particularly vulnerable to compensatory gratification through immersive short-video platforms such as TikTok. However, how paternal and maternal controls are jointly configured within this environment and how these specific family stressors are associated with PSVU remain underexplored. Investigating these dual-parent configurations and symptom-level networks within a Chinese sample not only broadens the cultural generalizability of developmental psychopathology but also provides crucial insights into how culturally embedded family risks uniquely shape the structural dynamics of adolescent digital addiction.

To address these gaps, this study adopted a two-stage design integrating person-centered and network-analytic approaches. First, LPA was used to identify heterogeneous subgroups based on the joint distribution of PPC, MPC, and PSVU in a sample of Chinese adolescents. Second, psychometric network analysis was applied to theoretically selected profiles, using a network comparison test (NCT) to examine whether profiles differing in PSVU outcomes also exhibited distinct dimension-level associative structures, and if so, where these differences lay (van Borkulo et al., 2023).

## 3. Methods

### 3.1. Participants and Procedure

The initial sample comprised 2725 students recruited from three middle schools in China. Prior to data analysis, 18 participants were excluded because of careless responses or failure to meet the developmental inclusion criteria (i.e., outside the target 12–16 age range or grade 1–2 cohort), resulting in a valid analytic sample of 2707 adolescents. Finally, 73 participants were excluded because of uniform response patterns or study attrition, resulting in a final analytical sample of *N* = 2634. The final sample consisted of 1296 boys (49.2%) and 1338 girls (50.8%) with a mean age of 13.59 years (*SD* = 0.75, range 12–16 years). Participants were distributed across Grade 1 (*n* = 1464, 55.6%) and Grade 2 (*n* = 1170, 44.4%) schools, and across three schools: School 1 (*n* = 734, 27.9%), School 2 (*n* = 1275, 48.4%), and School 3 (*n* = 625, 23.7%). This study was approved by the Institutional Review Board of the corresponding author’s affiliated institution and was conducted in accordance with the Declaration of Helsinki. Prior to data collection, written informed consent was obtained from all participants and their caregivers. The investigation was conducted in computer science classrooms with trained school counselors serving as the principal experimenters. Participants completed the assessments on computers, and data were collected via an online survey platform.

### 3.2. Measures

**Parental Psychological Control.** PPC and MPC were assessed using the four-item psychological control subscale derived from the Chinese Parent–Child Subsystem Quality Scale (CPCSQS; Shek & Law, 2016). Originally conceptualized by Barber (1996) to capture parenting behaviors that intrude upon adolescents’ emotional development, Shek (2006) translated, culturally adapted, and validated the construct for Chinese adolescents. A sample item is *“My father (my mother) always wants to change my thoughts or feelings.”* The CPCSQS has demonstrated robust factorial validity, strong internal consistency, and age appropriateness in numerous studies involving Chinese secondary school students (Shek & Law, 2016; Shek & Zhu, 2019). In the present sample, internal consistency was good for both PPC (Cronbach’s α = 0.882) and MPC (α = 0.896).

**Problematic short-video use.** PSVU was assessed using a 20-item short-video dependence scale comprising four subscales (A. Jiang et al., 2025): withdrawal and tolerance (5 items), preoccupation and persistence (5 items), damage and hindrance (5 items), and deception and escape (5 items), rated on a 5-point Likert scale. A sample item is, *“I have tried to spend less time watching short videos, but I can’t seem to do it.”* PSVU was assessed broadly across short-video platforms rather than restricted to any specific platform. Examples of widely used short-video applications in China, such as Douyin and Kuaishou, were provided to illustrate the types of platforms covered by the measure and facilitate participants’ understanding. Internal consistency was excellent for the total scale (α = 0.957) and good to excellent for all four subscales: preoccupation and persistence (α = 0.887), withdrawal and tolerance (α = 0.912), damage and hindrance (α = 0.900), and deception and escape (α = 0.877).

### 3.3. Data Analysis Strategy

To determine whether PPC and MPC were appropriately treated as separate rather than combined indicators, measurement invariance between the two scales was examined using a sequential confirmatory factor analysis approach with robust maximum likelihood estimation. A two-factor oblique model was specified, with four PPC items loaded on one factor and four MPC items loaded on a parallel factor. The configural model, in which all parameters were freely estimated, showed acceptable structural fit (*χ*^2^(19) = 764.33, *p* < 0.001, CFI = 0.890, RMSEA = 0.122, 90% CI [0.117, 0.127], SRMR = 0.040). Metric invariance was not supported (ΔCFI = −0.016, ΔRMSEA = −0.003), indicating that factor loadings could not be constrained to equality across parental targets without a meaningful reduction in fit. Only configural invariance was supported, meaning that the parallel factor structure was replicated across both parental versions, but that the item loadings and intercepts were not strictly equivalent. All standardized factor loadings exceeded 0.79 across models. The latent correlation between PPC and MPC was 0.812. The Pearson correlation coefficient between composite scores was *r* = 0.724, *p* < 0.05, 95% CI [0.706, 0.742]. These findings support the use of PPC and MPC as distinct indicators in subsequent analyses. Although configural invariance was supported, metric invariance was not, indicating that the two scales did not have equivalent factor loadings across the parental targets. Accordingly, PPC and MPC were entered as separate indicators in the LPA rather than being combined into a single composite.

A person-centered LPA was conducted to identify subgroups of adolescents exhibiting distinct multivariate configurations of PPC, MPC, and PSVU (Spurk et al., 2020). Six standardized indicators were specified: PPC, MPC, and four PSVU dimensions. PPC and MPC were entered as separate indicators rather than as a single composite parental control score because, despite their strong correlation (*r* = 0.81), fathers and mothers represent theoretically distinct socialization agents with potentially unique influences on adolescent development, consistent with parental role differentiation theory (Lamb, 2004).

Models specifying two to five profiles were estimated and compared using (a) Bayesian Information Criterion (BIC, lower values preferred), (b) classification accuracy via entropy (>0.70 considered acceptable) (Celeux & Soromenho, 1996), (c) the Lo–Mendell–Rubin adjusted likelihood ratio test (LMR-LRT) (Nylund et al., 2007), and (d) a minimum profile size criterion (≥5% of the total sample and *n* ≥ 30) to ensure substantive interpretability (Spurk et al., 2020).

Following profile identification, dimension-level network analyses were conducted to examine the associations underlying divergent PSVU outcomes. Analyses were directed toward theoretically informative subgroups, specifically those exhibiting elevated parental psychological control but markedly divergent PSVU, to examine how family-level exposures relate to these outcomes at the level of dimensional interconnection rather than mean differences alone. To ensure network estimation stability, six composite nodes were constructed, rather than entering all 20 individual items as separate nodes: PPC (mean of four items), MPC (mean of four items), and the four PSVU subscale scores. This decision was guided by the ratio of sample size to node count, as simulation studies have demonstrated that Extended Bayesian Information Criterion Graphical Least Absolute Shrinkage and Selection Operator (EBICglasso) can accurately recover network topology and effectively suppress false positive edges in networks with 10 or fewer nodes when the sample size is at or above 100 150 (Epskamp et al., 2018; van Borkulo et al., 2023). Gaussian Graphical Models were estimated for the targeted profiles using regularized partial correlation networks via the graphical LASSO algorithm with EBICglasso (tuning parameter = 0.5), implemented in the *Bootnet 1.9.1* package (Epskamp et al., 2018; Friedman et al., 2008). Network stability was assessed using non-parametric bootstrapping (1000 iterations) for edge-weight confidence intervals and case-dropping bootstrapping (1000 iterations) to compute the centrality stability (CS) coefficient for node strength (Epskamp et al., 2018). Formal statistical comparisons between the selected profile-specific networks were conducted using the *NetworkComparisonTest* 2.2.3 package (1000 permutations; van Borkulo et al., 2023). The NCT evaluated (a) global strength invariance, (b) network structure invariance (omnibus test of maximum edge weight difference), and (c) eight theoretically pre-specified bridge edges connecting parental control nodes (PPC, MPC) to each PSVU dimension (Jones et al., 2021), with the Holm–Bonferroni correction applied for multiple comparisons.

The community structure for the bridge centrality analysis was specified a priori based on the theoretical distinctions between the variables. The PPC and MPC were assigned to one community representing the family socialization context, and the four PSVU dimension nodes (PREOC, WITHD, DAMAG, and DECEP) were assigned to a second community representing adolescent digital dependence behavior. This two-community structure reflects the conceptual distinction between family-level environmental variables and adolescent behavioral outcome dimensions, consistent with the theoretical framing of parental psychological control as a contextual predictor rather than a component of the dependence construct (Borsboom, 2017; Jones et al., 2021). Bridge strength was computed for each node using the bridge() function from the Networktools package (version 1.6.0; Jones et al., 2021), which quantifies the sum of the absolute edge weights connecting a given node to nodes in the opposite community. Eight theoretically pre-specified bridge edges connecting the parental control nodes (PPC and MPC) to each of the four PSVU dimensions were extracted from the NCT output. Holm–Bonferroni correction was applied to these eight comparisons using p.adjust(method = “holm”) to control the familywise error rate.

## 4. Results

### 4.1. Stage 1: Latent Profile Analysis

*Model Selection.* The fit indices for the two- to five-profile solutions are presented in Table 1. Although BIC continued to decrease through the five-profile solution (BIC = 37,068.01), the four-profile solution was selected based on the combination of selection criteria specified in Section 3.3, which demonstrated the most favorable balance of parsimony and classification quality (entropy = 0.852). All profiles exceeded the minimum size threshold (smallest profile *n* = 141, 5.3%), and the LMR-LRT supported a significant improvement over the three-profile solution. In the five-profile solution, two profiles did not meet the commonly recommended minimum threshold of 5% of the total sample for LPA (Profile 2: *n* = 110, 4.18%; Profile 4: *n* = 100, 3.80%; Nylund et al., 2007; Spurk et al., 2020). By contrast, all profiles in the four-profile solution comfortably exceeded this threshold, with the smallest profile comprising 141 participants (5.35%). Furthermore, the newly identified profile (*n* = 626, 23.77%), characterized by moderate parental control (z ≈ 0.23) and moderately elevated PSVU (z = 0.85–0.99), closely resembled the existing Average-Control, Moderate-Dependence profile and did not reveal a qualitatively distinct latent subtype. Therefore, the five-profile solution was not retained.

**Figure 1:**
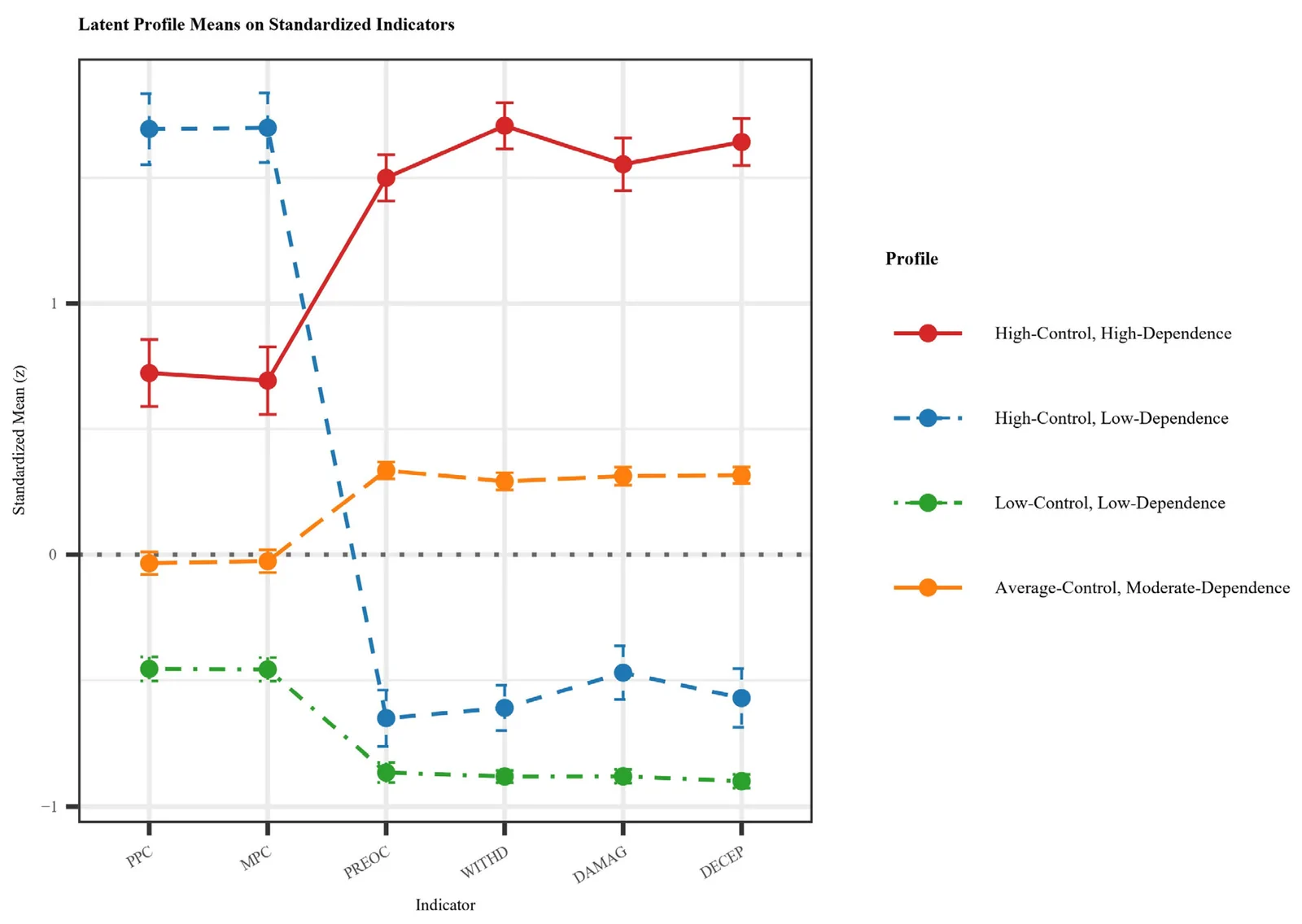
Standardized Means of Paternal Control, Maternal Control, and Problematic Short-Video Use Across the Four Latent Profiles. Note. PPC: paternal psychological control; MPC: maternal psychological control; PREOC = Preoccupation and Persistence; WITHD = Withdrawal and Tolerance; DAMAG = Damage and Hindrance; DECEP = Deception and Escape.

*Profile Descriptions.* Four latent profiles were identified (Table 1 and Figure 1). Consistent with the recommended practice for psychometric subgroup labeling, profiles were named descriptively based on observed indicator patterns rather than inferential labels, with the theoretical interpretation reserved for discussion.

Profile 1 (*High-Control, High-Dependence*; *n* = 306, 11.6%) was characterized by elevated PPC and MPC (PPC *z* = 0.72, MPC *z* = 0.69) co-occurring with elevated PSVU across all four dimensions (*z* range = 1.50–1.71). Profile 2 (*High-Control, Low-Dependence*; *n* = 141, 5.3%) showed high levels of parental psychological control (PPC *z* = 1.69, MPC *z* = 1.70) but markedly lower PSVU (*z* range = −0.65 to −0.47). Profile 3 (*Low-Control, Low-Dependence*; *n* = 916, 34.8%) was characterized by low parental psychological control (PPC *z* = −0.45, MPC *z* = −0.46) and low PSVU (*z* range = −0.90 to −0.87). Profile 4 (*Average-Control, Moderate-Dependence*; *n* = 1271, 48.2%, the largest subgroup) showed parental control levels close to the sample mean (PPC *z* = −0.03, MPC *z* = −0.03) accompanied by modestly elevated PSVU (*z* range = 0.29–0.33). The classification accuracy was high (entropy = 0.852), supporting the confidence in the profile assignment for subsequent analyses.

To test whether Profiles 1 and 2 differed in their primary family risk indicators, independent sample *Welch*’s *t*-tests were conducted to compare Profiles 1 and 2 on parental psychological control indicators. Profile 2 scored significantly higher than Profile 1 on both PPC (*M* = 3.05, *SD* = 0.60 vs. *M* = 2.37, *SD* = 0.84), *t*(366.49) = −9.80, *p* < 0.001, *M*_diff_ = −0.685, 95% CI [−0.822, −0.547]; *d* = −0.887, 95% CI [−1.094, −0.679], and MPC (*M* = 3.09, *SD* = 0.61 vs. *M* = 2.36, *SD* = 0.87), *t*(375.39) = −10.23, *p* < 0.001, *M*_diff_ = −0.729, 95% CI [−0.869, −0.589], *d* = −0.916, 95% CI [−1.124, −0.707]. Profiles 1 and 2 were above the sample mean for parental psychological control, with PPC and MPC z-scores above 0.65 and exceeding the scale midpoint of the original metric.

### 4.2. Stage 2: Dimension-Level Network Analysis

**Figure 2:**
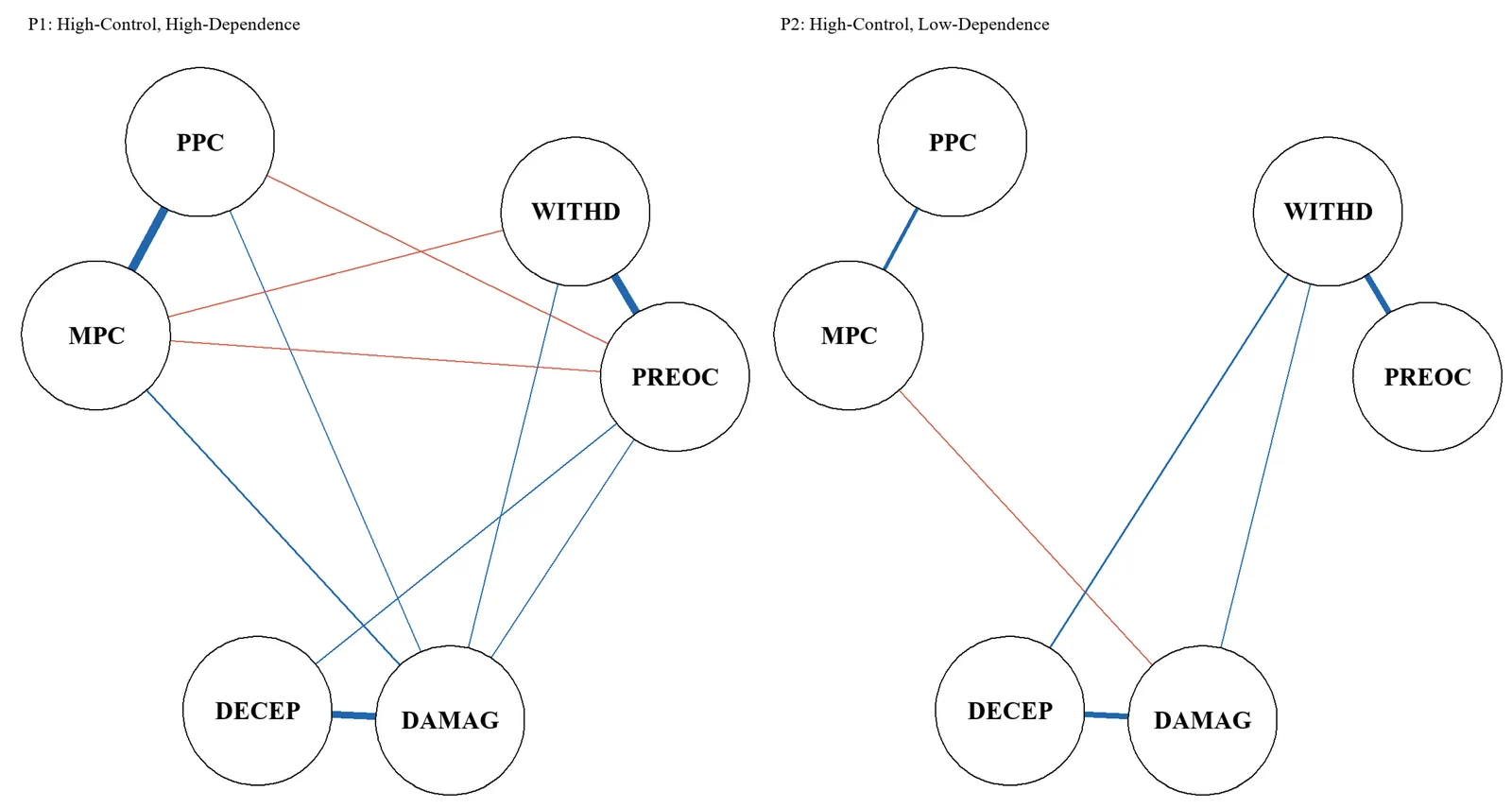
Regularized Partial Correlation Networks of Parental Psychological Control and Problematic Short-Video Use for the High-Control/High-Dependence Profile (**left**) and the High-Control/Low-Dependence Profile (**right**). *Notes*. PPC = Paternal Psychological Control; MPC = Maternal Psychological Control; PREOC = Preoccupation and Persistence; WITHD = Withdrawal and Tolerance; DAMAG = Damage and Hindrance; DECEP = Deception and Escape. Blue edges indicate positive partial correlations; red edges indicate negative partial correlations. Edge width reflects the magnitude of the partial correlation. Networks were estimated using the EBICglasso algorithm (tuning parameter = 0.5). Node positions are fixed across panels to facilitate visual comparison. P1 = High-Control, High-Dependence profile (*n* = 306); P2 = High-Control, Low-Dependence profile (*n* = 141).

Subsequent network analysis focused exclusively on Profiles 1 (High-Control, High-Dependence) and 2 (High-Control, Low-Dependence), the two profiles identified in Section 4.1 as both situated in the upper range of the parental psychological control distribution (PPC/MPC *z* > 0.65 in both profiles), yet exhibiting markedly divergent PSVU outcomes. As reported in Section 4.1, Profile 2 showed significantly higher absolute levels of parental psychological control than Profile 1 while exhibiting a substantially lower PSVU. This pairing provides a theoretically informative contrast for examining how profiles that differ in both control magnitude and dependence severity manifest distinct associative structures at the dimension level. Profiles 3 and 4 were excluded from the network analysis for two reasons. First, including all four profiles would introduce a multiple-comparison problem, substantially increasing the family wise error rate. Second, Profiles 3 (Low-Control, Low-Dependence) and 4 (Average-Control, Moderate-Dependence) represented markedly different levels of parental control exposure, such that any observed network differences would confound variations in control levels with variations in PSVU outcomes, limiting theoretically meaningful interpretation. Although Profile 2 showed significantly higher absolute levels of parental psychological control than Profile 1, both profiles were situated in the upper range of the control distribution, making their comparison more appropriate for examining how the network structure varies within a high-control context. Profile 1 showed elevated scores across all short-video use dimensions (PPC *M* = 2.37, MPC *M* = 2.36; PSVU dimensions *M*_range_ = 3.48–3.79), whereas Profile 2 showed markedly lower scores (PPC *M* = 3.06, MPC *M* = 3.09; PSVU dimensions M_range_ = 1.52–1.75). The skewness for all nodes was within acceptable limits (|skew| < 1.3), with the exception of Deception and Escape in Profile 2 (skewness = 1.27), noted as an interpretive caveat, given the restricted range of low scores within this profile. The regularized partial correlation networks for Profiles 1 and 2 are shown in Figure 2. The estimation accuracy for the network parameters was evaluated using nonparametric bootstrapping (1000 resamples via *bootnets*). In Profile 1, 3 of the 11 non-zero edges exhibited 95% bootstrapped confidence intervals, strictly excluding zero: the association between PPC and MPC (sample estimate = 0.716, 95% CI [0.612, 0.820]), PSVU Focus and Withdrawal (sample estimate = 0.597, 95% CI [0.489, 0.704]), and PSVU Impairment and Deception (sample estimate = 0.537, 95% CI [0.437, 0.637]). In Profile 2, 2 of the 6 nonzero edges maintained confidence intervals, excluding zero (focus-withdrawal and impairment-deception).

**Figure 3:**
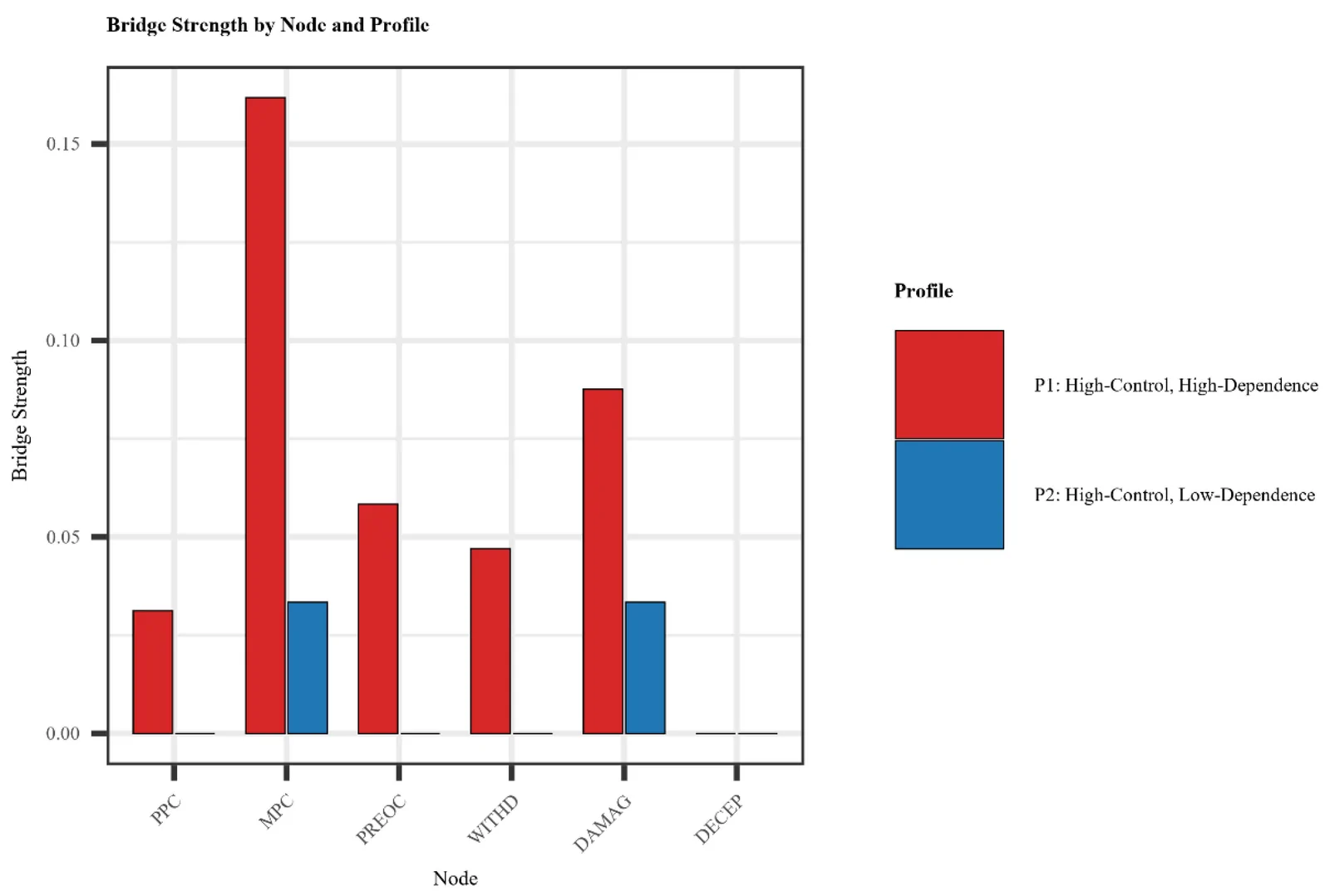
Bridge Strength Comparison. *Notes*. P1 = High-Control, High-Dependence Profile; P2 = High-Control, Low-Dependence Profile. PPC = Paternal Psychological Control; MPC = Maternal Psychological Control; PREOC = Preoccupation and Persistence; WITHD = Withdrawal and Tolerance; DAMAG = Damage and Hindrance; DECEP = Deception and Escape.

*Network Comparison Test*. A formal statistical comparison of the P1 and P2 networks was performed using NCT. The overall connectivity of the P1 network was significantly greater than that of the P2 network (global strength S = 0.854, *p* = 0.001), indicating that the High-Control, High-Dependence network exhibited stronger total connectivity than the High-Control, Low-Dependence network. The omnibus test of the maximum edge weight difference was also significant (*M* = 0.477, *p* = 0.001), indicating that the two networks differed in the specific pattern of dimension-level interconnections, and not merely in overall magnitude. The bridge strength values for all six nodes in both profiles are shown in Figure 3. In the P1 network, MPC showed the highest bridge strength (0.162) and PPC the lowest among parental control nodes (0.031), whereas bridge strength values in the P2 network were uniformly low across all nodes (range = 0.000–0.033; Table 2).

None of the eight pre-specified bridge edges linking the PPC/MPC to PSVU dimensions differed significantly between profiles after the Holm–Bonferroni correction (all corrected *p* = 1.00, except for MPCDamage and Hindrance, corrected *p* = 0.384; Table 3). This pattern suggests that the structural divergence between the two networks was not reflected in the detectable differences in the direct associations between parental control and individual PSVU dimensions.

**Figure 4:**
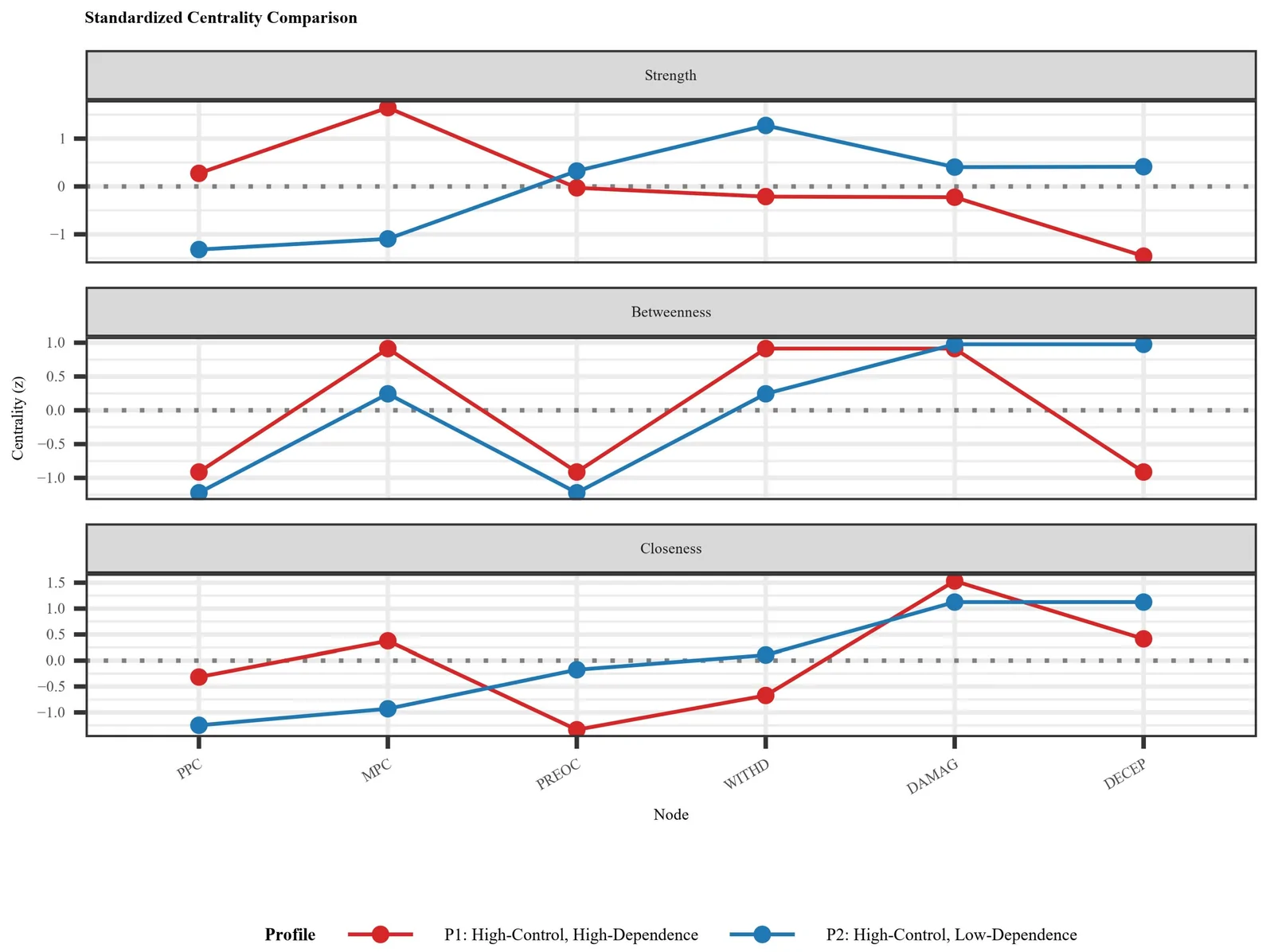
Standardized Node Centrality (Strength, Betweenness, and Closeness) in the Dimension-Level Networks of the High-Control/High-Dependence and High-Control/Low-Dependence Profiles. *Note.* The centrality indices were standardized (*z*-scores) within each profile network. P1 = High-Control, High-Dependence Profile; P2 = High-Control, Low-Dependence Profile. PPC = Paternal Psychological Control; MPC = Maternal Psychological Control; PREOC = Preoccupation and Persistence; WITHD = Withdrawal and Tolerance; DAMAG = Damage and Hindrance; DECEP = Deception and Escape.

*Centrality Stability.* The centrality stability was assessed using case-dropping bootstrapping. The CS coefficients for node strength were 0.363 for Profile 1 and 0.362 for Profile 2. Both values exceeded the minimum recommended threshold of 0.25, indicating acceptable stability for the node strength rankings in both networks (Epskamp et al., 2018). However, neither value reached the preferred benchmark of 0.50, indicating that centrality rankings should be interpreted with caution rather than as stable estimates of node importance. The six-node network estimated for Profile 2 (*n* = 141) satisfied the minimum sample size threshold recommended for stable EBICglasso recovery in networks with 10 or fewer nodes (Epskamp et al., 2018; van Borkulo et al., 2023), although the sample size approached this lower bound, and edge-weight estimates should accordingly be treated with caution. The standardized centrality values (strength, betweenness, and closeness) for both networks are shown in Figure 4.

Given that the CS coefficients fell short of the preferred stability threshold in both profiles, the following centrality patterns are reported only for descriptive purposes, and should not be interpreted as stable empirical findings. In the P1 network, MPC returned the highest node strength (strength = 1.646; bridge strength = 0.162), whereas PPC returned comparatively low values (strength = 0.274; bridge strength = 0.031). In the P2 network, the withdrawal and tolerance dimensions returned the highest node strength (strength = 1.275), whereas PPC returned the lowest (strength = −1.319). Given that the CS coefficients fell short of the preferred stability threshold and that the edge-weight estimates in Profile 2 carry greater uncertainty due to the smaller sample size, these centrality patterns are reported descriptively only, with the theoretical interpretation reserved for the Discussion Section.

## 5. Discussion

### 5.1. Overview of Findings

This study integrated person-centered and psychometric network approaches to examine the complex associative structures linking parental psychological control and adolescent PSVU. LPA identified four heterogeneous profiles, with a noteworthy contrast between two high-control profiles that diverged sharply in PSVU. Subsequent network analysis revealed that although these two profiles differed significantly in global connectivity strength and overall network structure, this divergence did not manifest in the direct bridge pathways linking PPC or MPC to specific PSVU dimensions.

### 5.2. Heterogeneous Profiles of Parental Psychological Control and Problematic Short-Video Use

The identification of four distinct latent profiles based on the joint distribution of parental psychological control and PSVU provides empirical support for the multifinality principle within DST (Cicchetti, 2006; Lerner, 2006). Profile 1 (high control, high dependence) and Profile 2 (high control, low dependence) were both in the upper range of the parental psychological control distribution, yet the profile with the highest control levels was associated with the lowest PSVU. This inverse pattern indicates that higher parental psychological control does not uniformly co-occur with greater dependence, and that the parental control–PSVU association may be more complex than linear models assume. One possible explanation lies in the SDT distinction between need-thwarting and behavioral compliance (Ryan & Deci, 2000; Soenens et al., 2008): under very high control, some adolescents may develop externally regulated compliance that suppresses problem behaviors in the short term without reflecting autonomous motivation or need satisfaction (Flamant et al., 2020, 2023). These findings are consistent with person-centered studies emphasizing heterogeneity in parenting effects (Efrati & Rosenberg, 2023; S. Fang et al., 2023; S. Xiong et al., 2023; T. Wang & Cheng, 2025; Zhou et al., 2022), and treating PPC and MPC as separate indicators offers a more granular view of family socialization than variable-centered approaches. Because the present study did not directly assess factors such as need frustration and autonomous motivation, future research is warranted to examine their relevance to these associations.

The largest profile, Profile 4 (average control, moderate dependence; 48.2%), also merits attention. These adolescents reported control levels near the sample mean alongside modestly elevated PSVU, suggesting that for a substantial portion of adolescents, PSVU is not associated with severe parental intrusion. Their moderate dependence may instead reflect factors in other ecological systems, such as peer norms, normative increases in reward sensitivity, or the algorithmically reinforced consumption cycles characteristic of short-video platforms (Casey & Caudle, 2013; Liao, 2024), consistent with the DST view that development is shaped by multiple interacting contexts (An et al., 2025; Lerner, 2006). This suggests that intervention frameworks centered exclusively on family dysfunction may be insufficient for the broader adolescent population, and that universal strategies may benefit from cultivating adolescents’ digital literacy and algorithmic awareness alongside family-level approaches (S. Li et al., 2026). Another finding was the highly parallel patterns of PPC and MPC across the four profiles, reflected in their closely aligned standardized means. This parallelism likely reflected both substantive and methodological factors. Parenting behaviors within families tend to covary due to shared environments, assortative mating, and co-parenting dynamics (Belsky & Jaffee, 2006; Simons & Conger, 2007), consistent with the high correlation between PPC and MPC (r = 0.72) and prior evidence that adolescents often perceived maternal and paternal psychological control as a coordinated family climate (Gao et al., 2025; Rogers et al., 2020; Soenens & Vansteenkiste, 2010). Shared informant perspectives and consistent response patterns across parallel items may have further contributed to this parallelism. Importantly, however, the similar profile means did not indicate that PPC and MPC were equivalent, as only configural invariance was supported and their item loadings and intercepts were not strictly equivalent. This distinction was further reflected in their differentiated node positions and bridge-strength patterns in the subsequent network analyses.

### 5.3. Dimension-Level Networks Structure Differences Between Key Profiles

The network comparison between Profiles 1 and 2 addresses a question that profile-level analysis alone cannot address. The LPA established that these two profiles differed in terms of PSVU outcomes and absolute levels of parental psychological control. However, mean-level differences between profiles do not reveal whether the internal dimension-level associative structure also differs or where such structural differences lie. Network analysis is precisely suited to this question as it estimates partial correlation structures among dimensions separately within each profile and enables a formal statistical comparison of these structures (Borsboom, 2017; Epskamp et al., 2018; van Borkulo et al., 2023). Bridge-edge analyses, which test whether specific associations between parental control and PSVU dimensions differ between profiles rather than overall connectivity, are also less susceptible to the circularity concerns inherent in two-stage designs (Jones et al., 2021). Together, these analyses provide structural information that neither profile membership nor between-profile mean comparisons can provide.

Responding to the core research aims, the NCT revealed that the dimension-level network in Profile 1 (high control, high dependence) exhibited significantly greater global connectivity strength and a distinct overall structure compared to Profile 2 (high control, low dependence). Consistent with the network theory of psychopathology, the more densely interconnected symptom system observed in Profile 1 was characterized by stronger connections among symptoms, providing a structural perspective on the higher level of behavioral dependence observed in this profile (Borsboom, 2017; J. Wang & Jia, 2026).

MPC returned the highest bridge strength among all nodes in the P1 network, suggesting a potentially stronger coupling with the PSVU dimensions than other nodes, whereas PPC occupied a more peripheral position. If replicated in larger samples, this pattern would be consistent with parental role differentiation theory, which proposes that mothers occupy a more proximal and emotionally regulative socialization role than fathers during adolescence (Lamb, 2004; Tang et al., 2024; Z. Xiong et al., 2026); whether MPC functions consistently as a more prominent connector than PPC remains a testable hypothesis for future work. In the P2 network, by contrast, bridge strength was uniformly low, with no node emerging as a prominent connector, suggesting that the family socialization and PSVU communities were more loosely coupled. Parental control nodes thus appeared more embedded within the broader network in P1 than in P2, a descriptive contrast that complements the non-significant NCT bridge-edge results. However, because the CS coefficients for both profiles fell below the preferred 0.50 threshold and Profile 2 had a relatively small sample size that approached the lower bound recommended for stable EBICglasso recovery, these patterns should be treated as preliminary rather than as stable estimates of node importance (Epskamp et al., 2018; van Borkulo et al., 2023). These patterns do not identify definitive intervention targets, and independent replication in larger samples is required before drawing inferences.

Given the analytical structure of the present study, the observed differences in network connectivity between profiles warrant careful interpretation. Latent profiles were constructed from the same six variables that were subsequently entered into the network analyses. Therefore, some portion of the global strength difference between Profiles 1 and 2 may reflect mean-level differences that define the profiles rather than qualitatively distinct associative dynamics. This is a structural feature of two-stage designs in which the grouping and outcome share a measurement basis, and we cannot fully rule this out with the present cross-sectional data. Although the difference in global strength reached statistical significance, this finding should not be considered direct evidence of stronger internal feedback loops or a more self-sustaining pattern of dependence. Establishing such interpretations requires longitudinal data that capture the temporal dynamics among symptom indicators, which the current design does not permit.

The bridge-edge comparisons, however, are less susceptible to circularity concerns, as they test whether specific associations between parental control and PSVU dimensions differ between profiles rather than overall connectivity. The null results indicate that the between-profile divergence in PSVU outcomes was not reflected in detectable differences in the specific associations linking parental control to individual PSVU dimensions. This non-significance should not be read as evidence of equivalence or as proof that parental control is unrelated to PSVU; it indicates only that the strength of these specific pathways did not differ significantly across profiles. This pattern is broadly consistent with SDT perspectives suggesting that parental psychological control may function as a distal contextual stressor associated with general need thwarting rather than as a proximal trigger of specific digital behaviors (Q. Q. Li & Liu, 2025; Ryan & Deci, 2017; Soenens et al., 2008). Overall, these network findings should be read as a source of hypotheses for future longitudinal work rather than as definitive evidence of causal or temporal dynamics.

### 5.4. Strengths, Limitations, and Future Directions

This study had several strengths. By distinguishing PPC and MPC as independent indicators within an integrated two-stage framework combining LPA and network analyses, it links the identification of heterogeneous subgroups to the examination of dimension-level associative structures within profiles, offering a more nuanced account of heterogeneity than either profile-level differences or variable-centered associations alone can provide.

Drawing on a large-scale sample of Chinese adolescents, the present findings suggest that family-related factors warrant consideration in understanding heterogeneity in PSVU across profiles, while avoiding a one-size-fits-all interpretation. Whereas much of the Western parenting literature conceptualizes autonomy-undermining control as psychologically intrusive, some parenting practices in Chinese families may be shaped by culturally meaningful constructs such as training (*guan*) and high parental devotion, which adolescents may experience as reflecting parental care rather than intrusion (Chao, 1994; Cheung & Pomerantz, 2011). This underscores the need to distinguish culturally normative parental involvement from intrusive psychological control that operates through guilt induction and love withdrawal (Pomerantz & Wang, 2009; Soenens & Vansteenkiste, 2010), as interventions indiscriminately targeting parental authority may encounter cultural resistance. Future research could directly examine the role of culturally specific Chinese parenting practices (e.g., *guan*) across distinct profile configurations. Given the cross-sectional design, modest sample size of Profile 2, and moderate centrality stability coefficients, the observed network metrics should not be directly translated into clinical intervention protocols; prospective longitudinal designs and randomized controlled trials are required to clarify the potential intervention value and causal mechanisms of these variables.

Despite these contributions, this study had several limitations that outline critical avenues for future research. First, the cross-sectional nature of the data restricts our capacity to draw definitive temporal or causal inferences; future research must employ longitudinal network approaches, such as cross-lagged panel network models, to rigorously establish causality and associative structures over time (Spiller et al., 2020). Second, the single-region sampling and relatively small sample size of Profile 2 limited the stability and generalizability of the network estimates. Consequently, the centrality rankings should be considered exploratory rather than definitive, particularly when drawing substantive conclusions regarding the importance of specific nodes. Future research should replicate these network structures in larger, independent, cross-regional, and cross-cultural samples, including clinical populations, to strengthen the robustness and generalizability of our findings (Z. Xiong et al., 2026). The relatively small sample size of Profile 2 represents a meaningful constraint on the precision of the network estimates. To mitigate this concern, we aggregated items into six composite nodes, applied EBICglasso regularization, and conducted nonparametric and case-dropping bootstrapping to assess edge weight precision and centrality stability. Although these safeguards improve estimation robustness, the network findings should be interpreted as exploratory and require replication in larger, representative samples. Another limitation concerns the concurrent assessment of all constructs through adolescent self-reports, which may increase the risk of common method bias. Future research should adopt longitudinal, multi-informant designs (e.g., integrating reports from parents, teachers, and adolescents) and objective measures of digital device use. Finally, to expand beyond the current focus on family microsystems, future research should incorporate peer nomination network data to thoroughly explore the potential buffering effects of extrafamilial social ecologies on adolescent PSVU.

### 5.5. Conclusions

Moving beyond traditional variable-centered paradigms, this study integrated LPA with psychometric network analysis to examine the unobserved heterogeneity and complex associative structures characterizing the co-occurrence of parental psychological control and adolescent PSVU. These findings indicate that the associations between elevated parental psychological control and PSVU outcomes are neither uniform across individuals nor linear in nature. Notably, the profile reporting the highest absolute parental psychological control exhibited the lowest PSVU, whereas structural differences between the high- and low-dependence profiles were reflected in the overall network connectivity patterns, rather than in the specific direct pathways linking parental control dimensions to individual PSVU dimensions. These findings underscore the value of integrating person-centered and network analytic approaches in research on family socialization and adolescent digital media use, and highlight the need for future precision-informed, culturally sensitive support strategies tailored to the specific risk configurations of diverse adolescent subpopulations.

## Figures and Tables

**Table 1:** LPA Model Fit Comparison.

Profiles	Model	LL	k	AIC	BIC	aBIC	Entropy	LMR-LRT	*p*	*n* min	**%min**
2	1	−20,135.25	19	40,308.50	40,420.15	40,359.78	0.837	4573.33	0.0099	1006	38.19
3	1	−19,167.22	26	38,386.45	38,539.23	38,456.62	0.864	1936.03	0.0099	298	11.31
4	1	−18,951.36	33	37,968.73	38,162.64	38,057.79	0.852	431.54	0.0099	141	5.35
5	1	−18,376.48	40	36,832.96	37,068.01	36,940.92	0.870	1149.95	0.0099	97	3.68

*Note*. LMR-LRT = Lo–Mendell–Rubin-adjusted likelihood ratio test; aBIC = sample size-adjusted Bayesian Information Criterion; *n* min = sample size of the smallest profile; %min = percentage of the total sample in the smallest profile.

**Table 2:** Node Centrality and Bridge Strength in the Dimension-Level Networks of the High-Control/High-Dependence and High-Control/Low-Dependence Profiles.

Group	Label	Betweenness	Closeness	Strength	Bridge Strength
P1	PPC	−0.913	−0.317	0.274	0.031
P1	MPC	0.913	0.378	1.646	0.162
P1	PREOC	−0.913	−1.335	−0.029	0.058
P1	WITHD	0.913	−0.673	−0.212	0.047
P1	DAMAG	0.913	1.533	−0.225	0.088
P1	DECEP	−0.913	0.415	−1.455	0.000
P2	PPC	−1.220	−1.249	−1.319	0.000
P2	MPC	0.244	−0.930	−1.095	0.033
P2	PREOC	−1.220	−0.176	0.323	0.000
P2	WITHD	0.244	0.105	1.275	0.000
P2	DAMAG	0.976	1.125	0.404	0.033
P2	DECEP	0.976	1.125	0.412	0.000

*Note*. PPC: paternal psychological control; MPC: maternal psychological control; PREOC = Preoccupation and Persistence; WITHD = Withdrawal and Tolerance; DAMAG = Damage and Hindrance; DECEP = Deception and Escape.

**Table 3:** Network Comparison Test: Global Strength, Structural Invariance, and Bridge Edge Differences Between Profiles.

Test	Statistic	*p* Value
Global strength invariance	0.854	0.001
Network structure invariance	0.477	0.001
*Bridge edge differences*		
PPC—PREOC	−0.021	1.000
PPC—WITHD	0.000	1.000
PPC—DAMAG	0.011	1.000
PPC—DECEP	0.000	1.000
MPC—PREOC	−0.038	1.000
MPC—WITHD	−0.047	1.000
MPC—DAMAG	0.110	0.384
MPC—DECEP	0.000	1.000

*Note*. PPC: paternal psychological control; MPC: maternal psychological control; PREOC = Preoccupation and Persistence; WITHD = Withdrawal and Tolerance; DAMAG = Damage and Hindrance; DECEP = Deception and Escape.

## Data Availability

Owing to privacy restrictions, data are only available from the corresponding author upon request.

## Abbreviations

The following abbreviations are used in this manuscript:

aBIC	Sample-Size Adjusted Bayesian Information Criterion
BIC	Bayesian Information Criterion
CS	Centrality Stability
DAMAG	Damage and Hindrance
DECEP	Deception and Escape
EBICglasso	Extended Bayesian Information Criterion Graphical Least Absolute Shrinkage and Selection Operator
LMR-LRT	Lo–Mendell–Rubin Adjusted Likelihood Ratio Test
LPA	Latent Profile Analysis
MPC	Maternal Psychological Control
NCT	Network Comparison Test
PPC	Paternal Psychological Control
PREOC	Preoccupation and Persistence
PSVU	Problematic Short-Video Use
SDT	Self-Determination Theory
WITHD	Withdrawal and Tolerance
